# Genetic predisposition to impaired metabolism of the branched chain amino acids, dietary intakes, and risk of type 2 diabetes

**DOI:** 10.1186/s12263-021-00695-3

**Published:** 2021-11-02

**Authors:** Weiqi Wang, Zengjiao Liu, Lin Liu, Tianshu Han, Xue Yang, Changhao Sun

**Affiliations:** National Key Discipline, Department of Nutrition and Food Hygiene, School of Public Health, Harbin Medical University, 157 Baojian Road, Harbin, 150081 People’s Republic of China

**Keywords:** Branched chain amino acid, Metabolism, Genetic risk score, Interaction, Type 2 diabetes, Chinese adult

## Abstract

**Background and objectives:**

Circulating branched chain amino acids (BCAAs) increase the risk of type 2 diabetes (T2D). The genetic variants in the BCAA metabolic pathway influence the individual metabolic ability of BCAAs and may affect circulating BCAA levels together with dietary intakes. So, we investigated whether genetic predisposition to impaired BCAA metabolism interacts with dietary BCAA intakes on the risk of type 2 diabetes and related parameters.

**Methods:**

We estimated dietary BCAA intakes among 434 incident T2D cases and 434 age-matched controls from The Harbin Cohort Study on Diet, Nutrition and Chronic Non-Communicable Diseases. The genetic risk score (GRS) was calculated on the basis of 5 variants having been identified in the BCAA metabolic pathway. Multivariate logistic regression models and general linear regression models were used to assess the interaction between dietary BCAAs and GRS on T2D risk and HbA1c.

**Results:**

Dietary BCAAs significantly interact with metabolism related GRS on T2D risk and HbA1c (*p* for interaction = 0.038 and 0.015, respectively). A high intake of dietary BCAAs was positively associated with diabetes incidence only among high GRS (OR 2.40, 95% CI 1.39, 4.12, *P* for trend = 0.002). Dietary BCAAs were associated with 0.14% elevated HbA1c (*p* = 0.003) and this effect increased to 0.21% in high GRS (*p* = 0.003). Furthermore, GRS were associated with 9.19 μmol/L higher plasma BCAA levels (*p* = 0.006, *P* for interaction = 0.015) only among the highest BCAA intake individuals.

**Conclusions:**

Our study suggests that genetic predisposition to BCAA metabolism disorder modifies the effect of dietary BCAA intakes on T2D risk as well as HbA1c and that higher BCAA intakes exert an unfavorable effect on type 2 diabetes risk and HbA1c only among those with high genetic susceptibility.

**Supplementary Information:**

The online version contains supplementary material available at 10.1186/s12263-021-00695-3.

## Introduction

Type 2 diabetes (T2D) has become an epidemic and imposes an enormous burden on health-care systems worldwide [1]. Insulin resistance is one of the primary and earliest characteristic feature of metabolic disorders which results to type 2 diabetes [2]. The pathogenesis of T2D are complex; both environmental and genetic elements can exert influence on the development of this disease [3, 4].

Branched chain amino acids (BCAAs, including leucine, isoleucine, and valine) are a group of essential amino acids playing an important role in protein synthesis and glucose metabolism and are associated with insulin resistance in type 2 diabetes and obesity [5–7]. Evidence suggests that the increased plasma BCAA levels can predict impaired insulin signaling and the development of T2D. Circulating BCAA levels showed a positive correlation with various clinical parameters for T2D, such as homeostasis model assessment for insulin resistance, HbA1c, and fasting blood glucose [8–10].

However, researches about the relationship between dietary BCAAs and T2D risk showed conflicting results. Dietary BCAAs showed a positive association with T2D in three cohorts of the US and the Women’s Health Initiative study, but negative in Japanese women [11–13]. Circulating BCAA levels modestly correlated with dietary intakes since they were affected by the process of BCAA intake and metabolism simultaneously [5]. On this basis, taking dietary intakes and the individual metabolism ability of BCAAs into account may clarify the effect of dietary BCAAs on T2D more accurately.

Recently, the huge increase in genome-wide association study (GWAS) has generated extensive knowledge about genetic variants in the BCAA metabolic pathway, which influence circulating BCAA levels and can be used to characterize an individual’s genetic predisposition to impaired BCAA metabolism ability [14]. In the present study, we used the genetic risk score (GRS) of the common variants in the BCAA metabolic pathway to determine whether individual metabolism ability will modify the association between dietary BCAAs and T2D risk.

## Methods

### Study population

The Harbin Cohort Study on Diet, Nutrition and Chronic Non-Communicable Diseases is a prospective cohort study of 9734 Chinese people aged 20–74 years at the study initiation in 2012. Information about demographic characteristics, dietary habits, and lifestyle was collected by trained health-care staff using a structured questionnaire and a physical examination was conducted at the same time. The details have been previously described in a previous study [15]. After excluding those who had T2D, deficient dietary data, and/or blood samples, implausible energy intake values (men > 16744 or < 3348 kJ/day, women > 14651 or < 2093 kJ/day) at the baseline survey and those who lack anthropometric measures in follow-up, 4964 participants remained in the cohort, including 434 new diagnosed diabetes cases. The cases were matched to 434 non-diabetic control subjects on age and sex.

This cohort study was reviewed by the institutional review boards of all institutes and was conducted in accordance with the Declaration of Helsinki. Written consent was obtained from all participants.

### Questionnaire survey

A food frequency questionnaire (FFQ) was taken at baseline to estimate long-term habitual diet, including 103 food items from 14 food groups (rice, wheat-containing foods, potato and its products, beans and their products, vegetables, fruits, livestock and its products, poultry and its products, dairy and its products, eggs and their products, fish and its products, snacks, beverages, and ice cream). The validity and reliability of the FFQ have been assessed in a previous study [16]. The Chinese Food Composition Tables were applied to calculate intakes of isoleucine (in mg/day), leucine (in mg/day), valine (in mg/day), total BCAAs (in mg/day), and protein (in g/day). They were calculated as:

95.$$ 95. $$ $$ \mathrm{Isoleucine}=\mathrm{rice}\ast 192.5+\mathrm{wheat}\ast 159.33+\mathrm{potato}\ast 54.17+\mathrm{beans}\ast 184.44+\mathrm{vegetable}\ast 29.89+\mathrm{fruit}\ast 9.4+\mathrm{livestock}\ast 297.09+\mathrm{poultry}\ast 371.25+\mathrm{milk}\ast 56.87+\mathrm{eggs}\ast 308.5+\mathrm{fish}\ast 375.2+\mathrm{snacks}\ast 123.25+\mathrm{beverage}\ast 8.5+\mathrm{ice}\ \mathrm{cream}\ast $$

201.5.$$ 201.5. $$ $$ \mathrm{Leucine}=\mathrm{rice}\ast 421.75+\mathrm{wheat}\ast 293.67+\mathrm{potato}\ast 78.67+\mathrm{beans}\ast 330.39+\mathrm{vegetable}\ast 44.90+\mathrm{fruit}\ast 15.43+\mathrm{livestock}\ast 536.23+\mathrm{poultry}\ast 652+\mathrm{milk}\ast 108.51+\mathrm{eggs}\ast 516.5+\mathrm{fish}\ast 665.7+\mathrm{snacks}\ast 213.17+\mathrm{beverage}\ast 16.55+\mathrm{ice}\ \mathrm{cream}\ast $$

$$ \mathrm{Valine}=\mathrm{rice}\ast 216.5+\mathrm{wheat}\ast 178.17+\mathrm{potato}\ast 77.33+\mathrm{beans}\ast 193.89+\mathrm{vegetable}\ast 36.78+\mathrm{fruit}\ast 14.75+\mathrm{livestock}\ast 322.32+\mathrm{poultry}\ast 402.5+\mathrm{milk}\ast 68.39+\mathrm{eggs}\ast 317.17+\mathrm{fish}\ast 425.9+\mathrm{snacks}\ast 153.08+\mathrm{beverage}\ast 20.32+\mathrm{ice}\ \mathrm{cream}\ast 140.5. $$

Total BCAAs = isoleucine + leucine + valine.

$$ \mathrm{Protein}=\mathrm{rice}\ast 1.30+\mathrm{wheat}\ast 3.98+\mathrm{potato}\ast 0.63+\mathrm{beans}\ast 4.53+\mathrm{vegetable}\ast 1.15+\mathrm{fruit}\ast 0.42+\mathrm{livestock}\ast 6.23+\mathrm{poultry}\ast 8.80+\mathrm{milk}\ast 1.40+\mathrm{eggs}\ast 6.70+\mathrm{fish}\ast 8.78+\mathrm{snacks}\ast 1.79+\mathrm{beverage}\ast 0.25+\mathrm{ice}\ \mathrm{cream}\ast 3.57. $$

Lifestyle and physical data were collected meanwhile. Exercise regularity was defined as recreational or sport physical activity performed at least 30 min for three or more days per week. The level of education was classified into no formal education, elementary school, middle/high school, technical school/college, postgraduate degree, or above. Current smokers were defined as those who have smoked at least 100 cigarettes in a lifetime and smoke every day or some days now. Current drinkers were defined as those who consumed ≥ 1 alcoholic drink each month in the past 12 months before the survey. Family history of diabetes was defined as those whose first-degree relatives suffered from diabetes. The presence of coronary heart disease at baseline was collected by using a structured questionnaire [17].

### Anthropometric measurements

Anthropometric indices were measured by trained medical workers according to a standard protocol. Weight, height, and waist circumference were measured in light clothing and without shoes to the nearest 0.1 kg and 0.1cm, respectively. Blood pressures were measured 3 times on the right arm of each participant after a 10-min rest, and the mean values were used for analysis. BMI was calculated as weight divided by the square of the height in meters.

### Biochemical analyses and outcome measures

An oral glucose tolerance test was carried out for each cohort participant according to the World Health Organization guidelines. Fasting and postprandial blood samples (fasting over 10 h and 2 h after drinking 75 g of glucose containing water) were collected for biochemical assessment. After collection, plasma samples were kept in a portable, insulated bag with ice packs (at about 0–4 °C) and were processed within 6 h for long-term storage at − 80 °C. Fasting blood glucose and 2-h glucose was measured quantitatively with an auto-analyzer (Hitachi 7100 Auto-analyzer, Tokyo, Japan).

Based on the oral glucose tolerance test, T2D cases were defined as fasting blood glucose ≥ 7.0 mmol/L and/or 2-h glucose ≥ 11.1 mmol/L or someone who self-reports T2D and takes control measures.

### DNA extraction, genotyping, and genetic risk score calculation

Peripheral venous blood samples were collected from each participant. Genomic DNA was extracted from peripheral blood leukocytes on a Tecan Freedom EVO platform (Tecan, Switzerland) using the Mag-Bind Blood DNA Kit (CWBIO, China). Single nucleotide polymorphism (SNP) genotyping work was performed using a custom-by-design 48-Plex SNPscanTM Kit (Cat#: G0104; Genesky Biotechnologies Inc., Shanghai, China). The SNPs we selected from GWAS were common variants that have been identified in the BCAA metabolic pathway and associated with circulating BCAA levels and had to meet the following criteria: fitting Hardy-Weinberg equilibrium, minor allele frequency exceeding 0.05, genotyping success rates exceeding 95%, and the genotyping concordance of replicated quality-control samples exceeding 95% in the replicated quality-control samples (10%). On this basis, 5 SNPs were selected to calculate GRS. The details of these SNPs are shown in Supplemental Table 1. We used a weighted method to calculate the genetic risk score on the basis of the 5 SNPs. Each SNP was recoded as 0 for homozygous for the non-effect allele and 1 for heterozygous and homozygous for the effect allele, and each SNP was weighted by its relative effect size (*β* coefficient) on plasma BCAA levels obtained from the previous genome-wide association study [18]. We calculated the genetic risk score by using the equation: GRS = (β_1_×SNP_1_+*β*_2_×SNP_2_+…+*β*_5_×SNP_5_) × (5/sum of the *β* coefficients), where *β* is the *β* coefficient for each individual SNP, and SNP_i_ is the numeric value of corresponding SNPs. The genetic risk score ranges from 0 to 5, with higher scores indicating a higher genetic predisposition to BCAA metabolism disorder [19]. In cohorts where genotypes were directly assessed, scores for individuals with missing genotypes were standardized to those with complete data, assuming that the missing genotypes were not related to disease status.

### Statistical analyses

BCAA intakes (sum of leucine, isoleucine, and valine intakes) and total protein intake were highly correlated (total BCAAs: *r* = 0.953, *P* < 0.0001) in partial correlation analysis after adjusting for sex, age, body mass index, waist circumference, physical activity, current smoking, current drinking, diabetes treatment, cardiovascular disease, fruit intakes, poultry intakes, and total energy intakes. Therefore, BCAA intakes were expressed as a percentage of total protein intake to reduce the likelihood of multicollinearity [20]. Dietary BCAAs and GRS were shown in classified mode, which were classified into 3 groups and 2 groups, respectively.

Odds ratios (ORs) and 95% CIs were calculated using the logistic regression model. The matching pairs were broken in subgroup stratified analyses. Using conditional analysis on matching factors may lead to loss of data [21]. Therefore, we used multivariable unconditional logistic analysis models to assess dietary BCAAs and T2D risk according to genetic risk subgroups. We tested interactions of the dietary BCAAs with genetic risk score on T2D risk by including the respective interaction terms in the models (for example, dietary isoleucine × genetic risk score), with the main effects included in the models as well. For the outcome of HbA1c and circulating BCAAs, we changed the model into multivariable linear regression models to calculate the effect size on HbA1c and circulating BCAAs as well as generalized linear models to measure the difference among groups and analyzed in the same way. In the multivariable analyses, we adjusted for covariates, including sex, age, body mass index, waist circumference, physical activity, current smoking, current drinking, diabetes treatment, cardiovascular disease, fruit intakes, poultry intakes, and total energy intakes. Analyzing the relationship between GRS and circulating BCAAs was further adjusted for fasting glucose, fat intakes, and animal protein intakes and corresponding dietary BCAAs as absolute intakes. When the missing data for covariates were fewer than 5%, we replaced the missing values with the median values.

We compared the characteristics at baseline for the Harbin Cohort Study and nested case-control study with a general linear model for continuous variables and chi-square tests for categorical variables. We used the SPSS software, version 25.0, for statistical analyses, and a two-sided *P* value < 0.05 was considered statistically significant.

## Result

### Participant characteristics

Total of 434 age- and sex-matched case-control couples were identified during the follow-up of the Harbin Cohort Study. The baseline characteristics of the nested case-control study population by dietary BCAA intakes are presented in Table 1. Participants with a higher dietary BCAA intake had higher BMI, waist circumferences, and energy intake, more likely to be current smokers, drinkers, and suffer T2D. Furthermore, those with higher genetic susceptibility of impaired BCAA metabolism were more likely to be accompanied with diabetic family history (Supplementary Table 2).

**Table 1:** Characteristics on baseline of nested case-control participants by dietary intake of total branched chain amino acids

	Total BCAA intakes (mg/g protein)			*P* value*
	T1 (*n* = 289)	T2 (*n* = 290)	T3 (*n* = 289)	
T2D cases, %	130 (45.1)	139 (47.9)	164 (56.7)	0.014
Age (years)	52.5 (8.3)	50.8 (8.4)	52.0 (9.5)	0.06
Male [*n*, (%)]	71 (24.7)	112 (38.6)	151 (52.2)	< 0.001
BMI (kg/m^2^)	25.1 (0.2)	25.1 (0.2)	25.8 (0.2)	0.041
Waist circumferences (cm)	85.6 (0.6)	87.1 (0.6)	89.9 (0.6)	< 0.001
Current smokers [*n*, (%)]	36 (12.5)	57 (19.7)	62 (21.5)	0.020
Current drinkers [*n*, (%)]	91 (31.6)	114 (39.3)	126 (43.6)	0.011
Exercised regularly [*n*, (%)]	148 (51.4)	122 (42.1)	137 (47.4)	0.079
Energy intake(kcal/day)	2199 (50.4)	2345 (50.2)	2600 (50.4)	< 0.001
Family history of T2D [*n*, (%)]	46 (16.1)	44 (15.2)	47 (16.3)	0.933
Hypertension [*n*, (%)]	74 (25.9)	57 (19.7)	79 (27.4)	0.074
Hyperlipid [*n*, (%)]	70 (24.5)	69 (24.0)	81 (28.4)	0.418
Coronary [*n*, (%)]	70 (24.3)	51 (17.6)	60 (20.8)	0.139
Dietary BCAAs (mg/g protein)				
Total BCAAs	174 (1.3)	200 (1.3)	253 (1.3)	< 0.001
Isoleucine	44.0 (0.3)	50.1 (0.3)	62.0 (0.3)	< 0.001
Leucine	79.7 (0.7)	93.4 (0.7)	121 (0.7)	< 0.001
Valine	49.8 (0.3)	56.5 (0.3)	70.0 (0.3)	< 0.001
Circulating BCAAs (μmol/L)				
Total BCAAs	11.8 (0.07)	11.9 (0.07)	11.9 (0.07)	0.23
Isoleucine	4.8 (0.05)	4.9 (0.05)	4.9 (0.05)	0.86
Leucine	3.9 (0.05)	3.9 (0.05)	4.0 (0.05)	0.25
Valine	9.9 (0.07)	10.1 (0.07)	10.1 (0.07)	0.21
Protein (g/day)	73.2 (2.1)	64.0 (2.1)	51.0 (2.1)	< 0.001
Fat (g/day)	77.8 (1.8)	77.1 (1.8)	72.0 (1.8)	0.049
Carbohydrate (g/day)	314 (8.4)	354 (8.3)	431 (8.4)	< 0.001
Fast glucose (mmol/l)	5.6 (0.1)	5.4 (0.1)	5.7 (0.1)	0.40

Data represent the mean ± SD or number (%)

*BCAAs*, branched chain amino acids; *BMI*, body mass index; *T2D*, type 2 diabetes

*P values were calculated by using the general linear model for continuous variables and chi-square tests for categorical variables

### Interactions between the GRS and dietary BCAA intakes on incident type 2 diabetes

Since high BCAA intake has been associated with increased incident T2D risk in earlier studies and our previous study [22], we analyzed the effect of BCAA intakes on the risk of type 2 diabetes among different GRS. Significant GRS-BCAA intakes interactions in relation to T2D risk were observed after multivariate adjustment(total BCAAs: *P* for interaction = 0.023; Table 2). When comparing the extreme tertiles of BCAA intakes separately within each GRS group, we found that higher BCAA intakes were more prominently associated with type 2 diabetes among high GRS (total BCAAs: OR 2.40, 95% CI 1.39, 4.12, *P* for trend = 0.002), but not among low GRS (total BCAAs: OR 0.93, 95% CI 0.53, 1.63, *P* for trend = 0.71).

**Table 2:** Tertiles of dietary BCAAs in relation to T2D according to the GRS of nested case-control participants

GRS	Dietary BCAAs (mg/g protein)			*P* for trend	*P* for interaction
	T1	T2	T3		
Total BCAAs					0.023
< 4(*n* = 424)	1	0.75(0.45–1.26)	0.93(0.53–1.63)	0.71	
≥ 4(*n* = 444)	1	1.69(1.00–2.84)	2.40(1.39–4.12)	0.002	
Isoleucine					0.024
< 4(*n* = 424)	1	0.82(0.49–1.39)	1.00(0.57–1.76)	0.93	
≥ 4(*n* = 444)	1	1.95(1.15–3.29)	2.55(1.48–4.38)	0.001	
Leucine					0.036
< 4(*n* = 424)	1	0.87(0.52–1.46)	0.98(0.56–1.72)	0.91	
≥ 4(*n* = 444)	1	1.67(0.99–2.81)	2.38(1.39–4.10)	0.002	
Valine					0.041
< 4(*n* = 424)	1	0.77(0.47–1.29)	0.89(0.51–1.56)	0.62	
≥ 4(*n* = 444)	1	1.43(0.85–2.39)	2.12(1.25–3.62)	0.005	

The multivariate logistic regression was used for estimation of ORs and 95% confidence interval (CI)

*BCAAs*, branched chain amino acids; *GRS*, genetic risk score; *T2D*, type 2 diabetes

Results were adjusted for age, sex, BMI, waist circumference, current drinkers, current smokers, physical activity, diabetes treatment, cardiovascular disease, fruit intakes, poultry intakes, and total energy intakes

### Interactions between the GRS and dietary BCAA intakes on HbA1c

We next performed interaction analyses of the quantitative traits of HbA1c that have been found to be associated with the variants in the BCAA metabolic pathway in GWAS. Dietary BCAA intakes significantly raised HbA1c in our study (effect size 0.14% (SE 0.047) for total BCAAs, *P* for trend = 0.003) (Supplementary Table 3). We then analyzed the association between BCAA intake and HbA1c levels among different GRS (Table 3). The association with elevated baseline HbA1c levels increased significantly with higher GRS (effect size for total BCAAs from 0.063% (SE 0.076) to 0.21% (SE 0.071) per tertile of BCAA intakes from low to high GRS, *P* for interaction = 0.038), and BCAA intakes were significantly associated with higher HbA1c levels only in high GRS individuals (*P* for trend = 0.003 for total BCAAs).

**Table 3:** Tertiles of dietary BCAAs in relation to HbA1c according to the genetic risk score in thirds of nested case-control participants

GRS	Dietary BCAAs(mg/g protein)			Effect size	*P* for trend	*P* for interaction
	T1	T2	T3			
Total BCAAs						0.038
< 4 (*n* = 424)	5.90(0.76)	5.82(1.11)	6.09(1.14)	0.10(0.076)	0.18	
≥ 4 (*n* = 444)	5.68(1.06)	6.01(0.74)*	6.13(1.27)*	0.21(0.071)	0.003	
Isoleucine						0.041
< 4 (*n* = 424)	5.89(0.76)	5.82(1.11)	6.10(1.14)	0.10(0.076)	0.13	
≥ 4 (*n* = 444)	5.68(1.06)	6.02(0.75)*	6.13(1.27)*	0.21(0.072)	0.003	
Leucine						0.050
< 4 (*n* = 424)	5.88(0.76)	5.83(1.11)	6.09(1.14)	0.10(0.075)	0.19	
≥ 4 (*n* = 444)	5.68(1.07)	6.01(0.74)*	6.13(1.27)*	0.21(0.071)	0.003	
Valine						0.114
< 4 (*n* = 424)	5.90(0.76)	5.83(1.10)	6.08(1.14)	0.11(0.077)	0.15	
≥ 4 (*n* = 444)	5.73(0.98)	5.96(0.86)	6.13(1.27)*	0.19(0.072)	0.020	

General linear model was used for estimation of mean (SD) for HbA1c (%) and linear regression model for *β* coefficient (SE)

*BCAAs*, branched chain amino acids; *GRS*, genetic risk score

Results were adjusted for age, sex, BMI, waist circumference, current drinkers, current smokers, physical activity, diabetes treatment, cardiovascular disease, fruit intakes, poultry intakes, and total energy intakes

∗Comparing to the lowest dietary intake group, *P* < 0.05

### Interactions between the GRS and dietary BCAA intakes on circulating BCAA levels

Because circulating BCAA levels were affected by dietary intakes and metabolism ability, we then focused on the interaction between the GRS and dietary BCAA intakes on circulating BCAA levels. GRS significantly interacted with BCAA intake in relation to the circulating BCAA level after multivariate adjustment (total BCAAs: *P* for interaction = 0.015; Table 4). Among all participants, GRS were associated with higher plasma BCAA levels (total BCAAs: effect size: 6.27μmol/L (SE 2.48), *P* for trend = 0.012) (Supplementary Table 4). These associations were strongest among the highest BCAA intake individuals (total BCAAs: effect size: 9.19μmol/L (SE 3.31), *P* for trend = 0.006), while GRS were not associated with plasma BCAA levels among the lowest and median BCAA intake individuals.

**Table 4:** The interaction between dietary BCAAs and GRS on the circulating BCAA level

Dietary BCAAs (mg)	Genetic risk score		Effect size	*P* for trend	*P* for interaction
	< 4 (*n* = 424)	≥ 4 (*n* = 444)			
Total BCAAs					0.015
T1 (*n* = 289)	139.95 (29.48)	139.53 (26.71)	0.55 (3.34)	0.87	
T2 (*n* = 290)	140.36 (24.87)	144. 80 (28.06)	1.93 (3.27)	0.56	
T3 (*n* = 289)	139.10 (29.80)	147.43 (29.10)*	9.19 (3.31)	0.006	
Isoleucine					0.054
T1 (*n* = 289)	23.80 (9.47)	24.38 (9.64)	0.61 (1.13)	0.59	
T2 (*n* = 290)	23.73 (8.94)	24.77 (9.55)	0.98 (1.17)	0.40	
T3 (*n* = 289)	24.06 (8.18)	24.45 (10.08)	0.93 (1.07)	0.39	
Leucine					0.64
T1 (*n* = 289)	15.47 (7.06)	15.42 (6.86)	− 0.55 (0.84)	0.52	
T2 (*n* = 290)	15.44 (5.55)	15.20 (6.52)	− 0.64 (0.77)	0.41	
T3 (*n* = 289)	16.54 (8.22)	16.42 (10.17)	0.70 (1.07)	0.52	
Valine					0.046
T1 (*n* = 289)	101.57 (26.03)	100.54 (21.70)	0.37 (2.91)	0.90	
T2 (*n* = 290)	100.49 (20.81)	104.34 (22.44)	1.99 (2.70)	0.46	
T3 (*n* = 289)	98.22 (21.91)	106.30 (21.51)*	7.34 (2.58)	0.005	

The general linear model was used for estimation of mean (SD) for circulating BCAAs (μmol/L) and linear regression model for *β* coefficient (SE)

*BCAAs*, branched chain amino acids

Results were adjusted for age, sex, BMI, waist circumference, current drinkers, current smokers, physical activity, diabetes treatment, cardiovascular disease, fasting glucose, fat intakes, animal protein intakes, fruit intakes, poultry intakes, and total energy intakes

∗Comparing to the lowest GRS group, *P* < 0.05

## Discussion

Although type 2 diabetes is thought to arise from a complex interplay between environmental factors and genetic predisposition, the specific interactions involved remain to be explored. Using the GRS constructed by the known variants in the BCAA metabolism pathway in our study, we observed the risk increase of type 2 diabetes with dietary BCAAs to be significantly amplified by high GRS. Analyses of HbA1c levels supported this observation as the dietary BCAAs were only associated with higher HbA1c levels among individuals with high GRS. Furthermore, we found that the association between metabolism-related GRS and circulating BCAA level was modified by BCAA intake; the positive association was more pronounced among those consuming higher BCAAs.

Several observational studies have researched the association between dietary BCAAs and type 2 diabetes but showed contradictory results [11–13]. Our results indicated that the effect of higher BCAA intakes was dependent on the individual genetic background of BCAA metabolism and the unfavorable effect was limited to those with a high genetic predisposition to impaired BCAA metabolism. This was in line with a recent report from the prospective longitudinal Nurses’ Health Study II cohort reporting that the association between BCAA intakes and type 2 diabetes risk was dependent on plasma concentrations; dietary BCAAs were associated with increased T2D risk only among those with high circulating levels while individuals with low circulating levels lacked such association [23]. Our analyses of HbA1c levels by GRS and BCAA intakes further supported such an interaction, as BCAA intakes were associated with higher HbA1c levels only among individuals with higher GRS.

Branched chain amino acids are vital nutrient signals that are essential for the maintenance of energy homeostasis with direct and indirect effects [24, 25]. Accumulating evidence supports the hypothesis that increased plasma concentrations of BCAAs contribute to raising negative feedback signaling to insulin receptor substrate 1 via persistent activation of mammalian target rapamycin complex 1 which promotes insulin resistance and impaired glucose metabolism [26, 27]. Dietary BCAA intakes and individual metabolism rate exert influence on circulating BCAA levels jointly. The metabolism of BCAAs primarily occurs in skeletal muscle transaminated through branched-chain-amino-acid transaminase and then dehydrogenizes through the branched-chain α-keto-acid dehydrogenase complex [28]. Its activity is inhibited by phosphorylation at a single site by branched-chain α-keto-dehydrogenase kinase while activated by the mitochondrial isoform of protein phosphatase 1K (PPM1K). Loss-of-function mutations in PPM1K in humans, and disruption of key BCAA metabolism in mice, exhibit a reduced expression of the metabolic-related enzyme, leading to increased plasma BCAA levels [5]. A Mendelian randomization study performed by Luca A et al. also demonstrated that specific gene variants in the BCAA pathway can influence individual metabolism ability and predict circulating BCAA levels [14]. Furthermore, our study found that the BCAA metabolism-related GRS may exert influence on circulating BCAA levels together with dietary BCAAs intakes, and the influences were more prominent when more BCAAs are consumed. Diet is the unique source of BCAAs and almost 80% of dietary BCAAs enter into the blood circulation [29]; therefore, heavy BCAA intakes will increase the demand of metabolism. So it is reasonable that the changes in plasma BCAA concentrations caused by genetic susceptibility of metabolism disturbance are magnified under conditions of high metabolism demand.

The results from some interventional studies have proposed that increasing dietary BCAA intakes have a positive effect on the parameters associated with obesity and T2D, such as improving glucose and cholesterol metabolism, maintaining lean body mass, and preventing insulin resistance by attenuating transcriptional coactivator peroxisome proliferator-activated receptor-γ coactivator, among apparently healthy subjects [30–32]. In rodent models, several feeding studies have shown the favorable effects of dietary BCAAs on glucose metabolism and insulin resistance [33]. But the rats feeding on a HF diet supplemented with BCAAs develop insulin resistance despite eating less food and gaining less weight than rats fed a HF diet alone [26]. Furthermore, obese mice always show a disruption of key BCAA metabolism leading to increased plasma BCAA levels; feeding them with a BCAA-restricted diet improves insulin sensitivity correspondingly [34, 35]. As a consequence, the effects of BCAAs on T2D and related parameters may depend on the individual metabolism ability of BCAAs. BCAA supplements in healthy individuals with intact metabolism could be well-tolerated by the reserve capacity of oxidation in the body that excess substrate can activate the related enzyme under normal conditions [36]. While in animals fed with a high-fat diet, or in obese individuals showing impaired amino acid oxidation, BCAA supplementation appears to be particularly harmful as it further increases the substrate load for mitochondrial oxidation causing mitochondrial stress and metabolic disturbances [37].

To the best of our knowledge, this is the first study to date to assess the interactions between genetic predisposition to BCAA metabolism disorder and dietary BCAAs on the incidence of T2D. Our data may provide novel information for the development of effective dietary recommendations strategies based on individual genetic backgrounds [38]. Strengths of our study include its prospective nested case-control design, which allows for the measurement of exposures before T2D diagnosis and mitigates the influence of the outcome and related treatments on participants’ dietary and metabolic profiles. However, several limitations of this study need to be considered. Firstly, using FFQ to assess long-term usual dietary intake may suffer measurement error; however, dietary measurement error may attenuate associations only and cannot explain the gene-diet interactions observed, assuming that errors occur independent of GRS. Secondly, the relatively small sample size of our study population may be underpowered for detecting modest interactions and the racially homogenous limited the generalizability of our findings to other ethnic groups. Replication of these findings in another cohort is required to enhance the validity of our results.

In conclusion, our study suggests that the genetic susceptibility of BCAA metabolism disturbance modifies the association of dietary BCAA intakes with T2D incidence and HbA1c levels. Heavy BCAA intakes showed unfavorable effects on the risk of T2D and HbA1c levels among genetically high risk of damaged metabolism population but not among those with low risk. Our novel findings question the current dietary recommendations which are based on population averages and contribute to support the notion of a personalized nutrition advice in preventing type 2 diabetes.

## Supplementary Information

**Additional file 1: Supplementary Table 1.** Characteristics of genetic variants associated with BCAA metabolism.

**Additional file 2: Supplementary Table 2.** Characteristics on baseline of nested case-control participants by by genetic risk score.

**Additional file 3: Supplementary Table 3.** Tertiles of dietary BCAAs in relation to HbA1c of nested case-control participants.

**Additional file 4: Supplementary Table 4.** Circulating level of BCAAs according GRS in thirds of nested case-control participants.

## Data Availability

All of the data are available with reasonable request from the corresponding author.

## Abbreviations

BCAAs: Branched chain amino acids
GRS: Genetic risk score
GWAS: Genome-wide association study
SNP: Single nucleotide polymorphism
T2D: Type 2 diabetes
